# Whole-Genome Sequencing and Comparative Genomic Analysis of Antimicrobial Producing *Streptococcus lutetiensis* from the Rumen

**DOI:** 10.3390/microorganisms10030551

**Published:** 2022-03-03

**Authors:** Isabela Maria Fernandes de Oliveira, Fernanda Godoy-Santos, Linda Boniface Oyama, Sofia Magalhães Moreira, Rodrigo Gonçalves Dias, Sharon Ann Huws, Christopher J. Creevey, Hilário Cuquetto Mantovani

**Affiliations:** 1 Departamento de Microbiologia, Universidade Federal de Viçosa, Viçosa 36570-900, MG, Brazil; isabelamfo@gmail.com (I.M.F.d.O.); f.godoysantos@qub.ac.uk (F.G.-S.); sofiamagmoreira@gmail.com (S.M.M.); rodrigo.dias@ufv.br (R.G.D.); 2 Institute for Global Food Security, School of Biological Sciences, Medical Biology Centre, Queen’s University Belfast, Belfast BT9 5DL, UK; l.oyama@qub.ac.uk (L.B.O.); chris.creevey@qub.ac.uk (C.J.C.)

**Keywords:** Nelore cattle, antimicrobial peptides, biosynthetic gene clusters, bacteriocins, CRISPR-Cas system, streptococci

## Abstract

Antimicrobial peptides (AMPs) can efficiently control different microbial pathogens and show the potential to be applied in clinical practice and livestock production. In this work, the aim was to isolate AMP-producing ruminal streptococci and to characterize their genetic features through whole-genome sequencing. We cultured 463 bacterial isolates from the rumen of Nelore bulls, 81 of which were phenotypically classified as being *Streptococcaceae*. Five isolates with broad-range activity were genome sequenced and confirmed as being *Streptococcus lutetiensis*. The genetic features linked to their antimicrobial activity or adaptation to the rumen environment were characterized through comparative genomics. The genome of *S. lutetiensis* UFV80 harbored a putative CRISPR-Cas9 system (Type IIA). Computational tools were used to discover novel biosynthetic clusters linked to the production of bacteriocins. All bacterial genomes harbored genetic clusters related to the biosynthesis of class I and class II bacteriocins. SDS-PAGE confirmed the results obtained in silico and demonstrated that the class II bacteriocins predicted in the genomes of three *S. lutetiensis* strains had identical molecular mass (5197 Da). These results demonstrate that ruminal bacteria of the *Streptococcus bovis*/*equinus* complex represent a promising source of novel antimicrobial peptides.

## 1. Introduction

The rumen ecosystem is colonized by a diverse microbial community, composed mainly of commensal anaerobic bacteria, archaea, protozoa, fungi, and viruses that are essential to the rumen function and the nutrition of the host [1]. Bacteria play a major role in the digestion of fibrous feedstuffs and some species of ruminal bacteria are a source of potent fibrolytic enzymes and metabolites of biotechnological interest, including antimicrobial peptides (AMPs) [2,3,4,5]. The capacity of rumen bacteria to produce antimicrobial compounds enhances their response to competitive challenges, enables occupancy of an ecological niche, and improves survival within this complex microbial community [6].

Previous studies indicated that the genera *Ruminococcus* [7], *Butyrivibrio* [8], *Enterococcus* [9], and *Streptococcus* [10,11] are frequently associated with the production of antimicrobial peptides [12,13]. The methods used for prospecting such molecules encompass both traditional culture-based approaches [14] as well as in silico analyses of biosynthetic gene clusters (BGCs) in rumen bacterial genomes using computational tools [6,12]. In a previous in silico study, we demonstrated that lanthipeptide BGCs are distributed in the genomes of ruminal streptococci, but antimicrobial activity was not investigated in the strains carrying the biosynthetic machinery [12]. In addition, the production of antimicrobial peptides within this group of ruminal bacteria has been largely underexplored, and traditional screening methods have not been matched with whole-genome sequencing data to expedite the identification of BGCs potentially responsible for the production of antimicrobial peptides and to expose genomic differences between ruminal streptococci showing antibacterial activity. Moreover, the antimicrobial peptides produced by ruminal streptococci also have applied interest since these molecules may represent a potentially useful alternative to manipulate ruminal fermentation and to control the growth of foodborne pathogens and food spoilage organisms [15].

In this work, we screened 463 bacterial isolates obtained from Nelore (*Bos indicus*) cattle feeding tropical forages, focusing on the selection of ruminal streptococci with anti-bacterial activity. The aim was to perform genome mining of biosynthetic gene clusters for bioactive compounds in strains producing diffusible inhibitory compound and to characterize, through comparative genomic analysis, the shared and unique genetic features between these strains. Here, we report the draft genome assemblies of five novel strains of *Streptococcus lutetiensis* isolated from the bovine rumen. Our analyses revealed that these strains harbor highly conserved putative BGCs associated with the production of antimicrobial peptides among ruminal streptococci. The ruminal *S. lutetiensis* strains with antibacterial activity shared many genomic features, including several genes associated with a less fastidious lifestyle compared to other non-ruminal streptococci. In addition, our analysis revealed unique protein sequences or genetic traits (e.g., CRISPR-Cas9 system) that were associated, or not, with the antimicrobial activity in each *S. lutetiensis* strain.

## 2. Materials and Methods

### 2.1. Isolation, Growth Conditions, and Maintenance of Microorganisms

The fistulated Nelore bulls were maintained in the facilities of the unit for teaching, research, and extension in beef cattle at the Universidade Federal located in the Southeast region of Brazil. Ruminal fluid (approximately 1 L) was collected from each animal between 3 and 4 h after the morning feed. Ruminal solids were immediately filtered through four layers of cotton gauze, stored in hermetically sealed bottles, and transported to the laboratory. The ruminal fluid was incubated at 39 °C for 30 min to allow feed particles to float, and samples were collected under anaerobic conditions from the middle of the flask and used for the isolation of ruminal streptococci. The flasks were then transferred to an anaerobic chamber (gases mixture 95% CO_2_ with 5% H_2_) for further manipulation. Briefly, rumen fluid (100 μL) from each sample was serially diluted into 900 μL of anaerobic 0.85% NaCl solution, and aliquots (100 μL) were streaked in duplicate onto anaerobic medium with the following composition (per liter): 292 mg of K_2_HPO_4_, 292 mg of KH_2_PO_4_, 480 mg of (NH_4_)SO_4_, 480 mg of NaCl, 100 mg of MgSO_4_·7H_2_O, 64 mg of CaCl_2_·2H_2_O, 600 mg of hydrochloric cysteine, 1000 mg of Trypticase, 500 mg of yeast extract, 400 mg of Na_2_CO_3_, 8000 mg of glucose [16] and 1.5% agar. The plates were incubated anaerobically at 39 °C for 24–48 h and colonies showing distinct phenotypic characteristics (differences in colony size, pigmentation, and morphology) were selected and sub-cultured from each plate into fresh anaerobic liquid medium. A total of 463 pure cultures were isolated and kept initially in 96-well polystyrene plates and sub-cultured every 48 h until they were evaluated for antimicrobial activity. Gram staining for morphological characterization, in addition to catalase test and analysis of fermentation end products using High-Performance Liquid Chromatography (HPLC) were performed with the isolates showing inhibitory activity before anaerobic stocks with 20% glycerol were prepared for long term storage at −80 °C.

### 2.2. Spectrum of Activity

The antimicrobial activity of the ruminal isolates against the target organisms was evaluated by deferred inhibition assays and agar well diffusion assays [17]. Deferred inhibition assays were performed using 1 μL of overnight culture from each producing strain on solid media. Plates were incubated anaerobically at 39 °C for 24 h to allow the growth of colonies and the inverted plates were exposed to chloroform vapors (99.8%, Acros Organics, Fair Lawn, NJ, USA) for 30 min to inactivate the cells on the agar surface. Next, the plates were overlaid with semi-solid media (0.85% agar, *w*/*v*) seeded with each of the target organisms. The plates were kept overnight at 4 °C and then were incubated at 37 °C, a temperature that allowed for the growth of the target organisms. The presence of clear zones of inhibition ≥2 mm surrounding the producer colonies was considered evidence of antimicrobial activity.

Agar well diffusion assays were also performed using cell-free supernatants harvested by centrifugation (8000× *g* for 10 min, room temperature) from ruminal isolates that were cultured (39 °C/24 h) in anaerobic media [18]. The target organisms were inoculated into melted solid media (1.5% agar, *w*/*v*) precooled to 50 °C and the mixture was rapidly transferred into sterile Petri plates, allowed to solidify and dry. Aliquots (25 microliters) of the cell-free supernatants were then added to the wells (5 mm in diameter) and allowed to diffuse for approximately 6 h at 4 °C. The plates were then incubated at 37 °C to allow the growth of the target organisms and the appearance of clear zones of inhibition (≥2 mm) on the target lawn around the wells was taken as indicative of antimicrobial activity.

All isolates (*n* = 463) were initially tested against four bacterial targets: *Enterococcus faecalis* ATCC 4083, *Escherichia coli* ATCC 10536, *Staphylococcus aureus* ATCC 29213 and *Lactobacillus paracasei* subsp. *paracasei* ATCC 335. A total of 106 ruminal isolates showing inhibition activity against at least two of these bacterial targets were selected for further investigation. The spectrum of activity of the 106 isolates was evaluated against the following additional bacterial targets: *Listeria monocytogenes* ATCC 7644, *Salmonella enterica* serovar *Typhimurium* ATCC 14028, *Proteus vulgaris* ATCC 13315, *Staphylococcus aureus* ATCC 25923, *Shigella flexneri* ATCC 12022, *Citrobacter freudii* ATCC 8090, *Klebsiella pneumoniae* ATCC 13882, and *Pseudomonas aeruginosa* ATCC 27853 and the drug-resistant pathogen *Acinetobacter baumanni* ATCC 19606 [19]. All indicators were cultured aerobically in BHI medium (Brain Heart Infusion Broth, Himedia, Mumbai, India) at 37 °C for 24 h. For the deferred inhibition assays, the target bacteria were cultured in BHI media for 24 h and diluted to a final concentration of 10^5^ CFU·mL^−1^. The experiments were performed in triplicate.

### 2.3. Genomic DNA Extraction and 16 rRNA Gene Sequencing

Isolates phenotypically characterized as streptococci that showed broader spectrum and exhibited higher activity against the bacterial targets evaluated in the current work were selected (*n* = 5) and subjected to genomic DNA extraction using the Wizard^®^ Genomic Purification Kit (Promega, Madison, WI, USA), according to the manufacturer’s specifications. DNA samples were analyzed on 0.8% agarose gels (Sigma-Aldrich, St. Louis, MO, USA) in 1X TAE buffer [Tris-HCl 40 mM, 20 mM acetic acid, and 1 mM EDTA (pH 8)], and were stained with ethidium bromide, 25 μg mL^−1^) [20]. Gels were visualized under UV light in the EagleEye™ image scanning system (Stratagene, Cedar Creek, TX, USA). DNA quantification was performed spectrophotometrically by measuring absorbance at 260 nm. Initial taxonomic characterization of the ruminal streptococci selected in this study was performed through sequencing of the 16S rRNA gene using the universal primers 27F (5′-AGAGTTTGATCMTGG-3′) and 1492R (5′-ACGGGCGGTGTGTRC-3′). The following conditions were used for each amplification cycle (total of 30 cycles) in a Biocycler MG96G thermocycler (São Paulo, Brazil): 94 °C for 3 min, 94 °C for 1 min, 50 °C for 1 min, 72 °C for 1.4 min, followed by a final step at 72 °C for 7 min. The reactions had approximately: 10 ng of genomic DNA, 5 μL of GoTaq reaction buffer (5×), 1.5 μL of MgCl_2_ (0.5 mmol L^−1^), 0.5 μL of dNTPs (2.5 mmol L^−1^), 1 μL of each primer (10 mmol L^−1^), 0.2 μL Taq DNA polymerase (Promega Corporation, Madison, WI, USA) for a reaction mixture of 25 μL. The PCR product was confirmed on a 1.5% agarose gel.

### 2.4. Genome Sequencing and Assembly

The genomes of the selected ruminal streptococci (*n* = 5) were sequenced using the Illumina HiSeq 2000 (Illumina Inc., San Diego, CA, USA) platform at MicrobesNG, Birmingham/United Kingdom. Raw reads were initially trimmed with Trimmomatic 0.30 using a 4-base sliding window and quality score of Q15 [21]. Genome assembly was performed using SPAdes version 3.7 [22]. The genome sequences were deposited in DDBJ/ENA/GenBank (https://www.ebi.ac.uk/ena/, accessed on 14 June 2021) under the following study accession code, PRJEB28470, and the following run accession codes: ERR5159231 (*Streptococcus lutetiensis* UFV09); ERR5159232 (*Streptococcus lutetiensis* UFV11); ERR5159233 (*Streptococcus lutetiensis* UFV58); ERR5159234 (*Streptococcus lutetiensis* UFV59), and ERR5159235 (*Streptococcus lutetiensis* UFV80).

The 16S rRNA gene sequences from each genome were BLAST searched against the 16S ribosomal RNA sequences (Bacteria and Archaea) database and RefSeq genome database of the National Center for Biotechnology Information (NCBI) (https://blast.ncbi.nlm.nih.gov/, accessed on 14 June 2021). Other *Streptococcus* 16S gene sequences showing identity ≥99.8% to 16S gene sequences from Global Rumen Census Project (GRC) [23] and identity ≤98% to 16S gene sequences from Human Microbiome Project (HM) [24] were selected from the NCBI database and used for comparative analyses. Multiple alignments of the 16S rRNA gene sequences were completed using RDP Aligner release 11 [25]. The 16S rRNA gene phylogenetic tree was reconstructed in FastTree v2.1 [26] (1000 replicates), using the Maximum Likelihood method. The tree was visualized and annotated in the Interactive Tree Of Life v5 (iTOL) (https://itol.embl.de, accessed on 14 June 2021) [27], and *Clostridium beijerinckii* NCIMB 8052 was chosen as the out-group to root the phylogenetic tree.

### 2.5. Annotation and Comparative Genomic Analysis

Assembled genomes were submitted to the comprehensive genome analysis service in the Pathosystems Resource Integration Center (PATRIC platform, https://www.patricbrc.org, accessed on 14 June 2021) [28] using default parameters. The genome sequences were annotated through the PATRIC platform based on the RAST tool (RASTtk) using the reference genetic code 11 (Translation table 11) used for Archaea and Bacteria [29]. The multidimensional omics data from each genome was visualized using the Circos approach [30]. A comparative proteomic analysis was performed in the PATRIC platform using bidirectional BLASTp among the genomes of the selected ruminal streptococci considering *S. lutetiensis* UFV59 as the reference strain in these analyses. The following default features were used: minimum sequence coverage of 30%, minimum identity of 10%, and BLAST E-value of 10^−5^.

### 2.6. CRISPR/Cas9 Characterization

CRISPR-Cas system elements detected in the genome of the *S. lutetienses* UFV80 in the PATRIC comprehensive genome analysis [28] were submitted to specific CRISPR analysis platforms. The prediction of CRISPR arrays of repeat-spacer units, and cas gene types (and subtypes) of predicted system(s) and anti-repeats (part of tracrRNA genes of type II CRISPR–Cas systems) were performed at the CRISPRone platform (https://omics.informatics.indiana.edu/CRISPRone/, accessed on 14 June 2021) [31] using default parameters.

The direct repeats (DR) consensus sequences were analyzed using default parameters of CRISPRmap (http://rna.informatik.uni-freiburg.de/CRISPRmap/Input.jsp, accessed on 14 June 2021) [32]. The potential target identification of CRISPR RNA was carried out on the CRISPRtarget platform (http://crispr.otago.ac.nz/CRISPRTarget/crispr_analysis.html, accessed on 14 June 2021) using the default parameters (gap open:-10; extend:-2; nucleotide match:1; mismatch:-1; E-value:1; word size:7) [33] and the following databases containing the target/protospacer sequences were selected: Genbank-Phage, Ref-Seq-Plasmid, IMG/VR, Ref-Seq Archaea, Genbank-Environmental, ACLAME, and HuVirDB [34]. Redundant spacers were removed during the analyses and a score cutoff of 20 was used to filter the results. Similar spacer sequences were searched in the Hungate genomes using a specific CRISPR spacer database generate in a previous study [35]. For this, the BLASTn-short was carried out using the Galaxy/Europe platform (https://usegalaxy.eu, accessed on 14 June 2021).

### 2.7. Genome Mining of Biosynthetic Gene Clusters

The five genome sequences of *Streptococcus* spp. strains isolated in the current study were mined for the presence of putative biosynthetic gene clusters potentially encoding antimicrobial compounds using antiSMASH 5.0 (https://antismash.secondarymetabolites.org/#!/start, accessed on 14 June 2021) [36]. The draft genomes were uploaded as a .fasta file and the biosynthetic gene clusters were annotated selecting the following default features: KnowClusterblast, SubClusterBlast, smCoGanalysis, and ActiveSiteFinder.

### 2.8. Characterization of Bacteriocin Precursor Peptides

Precursor peptide sequences belonging to the class II bacteriocins identified by antiSMASH 5.0 [36] were evaluated for the presence of conserved amino acid residues related to the bacteriocin leader and core sequences. This included the double glycine (GG) residues located at positions –1 and –2 relative to the core sequence, the pediocin box (YGNGVXC) in the N-terminal region of the core sequence, and the presence of cysteine residues indicative of disulfide bond formation [37,38,39]. For this, the precursor peptide sequences were subjected to BLAST-search in the Uniprot platform (https://www.uniprot.org/, accessed on 14 June 2021) [40]. When the identification of the precursor peptide sequences in the bacteriocin clusters by antiSMASH was uncertain, putative sequences were analysed by BLASTp search in the NCBI [41], and those showing a high score match to bacteriocin sequences were selected as the potential precursor peptides.

### 2.9. Ruminal Genomic Occurrence of Bacteriocin Precursor Peptides

All precursor peptide sequences identified in the genomes of the five *Streptococcus lutetiensis* strains were BLASTp searched against the annotated genomes of the Hungate1000 project [35] using default parameters on the Galaxy platform (https://usegalaxy.eu/, accessed on 14 June 2021) [42]. All subjected sequences listed in the BLASTp file were extracted from the compiled annotated fasta genome file by command line using Samtools v1.5 [43]. Query and subject sequences were aligned by Muscle v3.8.31 [44] and phylogenetic trees were constructed using the Maximum-Likelihood method by FastTree v2.1 [26] (1000 replicates). Trees were visualized and annotated in the Interactive Tree Of Life v5 (iTOL) (https://itol.embl.de, accessed on 14 June 2021) [27].

### 2.10. Bacteriocin Extraction, Purification and Activity

*Streptococcus lutetiensis* UFV9, UFV11 and UFV58 were grown in anaerobic medium (350 mL) at 39 °C for 48 h, as described above. *S. lutetiensis* UFV59 and UFV80 were not included in this analysis due to the lack of activity of the cultures grown in liquid media, under the conditions evaluated in this study. The cell-associated bacteriocins were extracted from the bacterial biomass harvested by centrifugation (8000× *g*, 10 min, 4 °C). The cell pellets were washed in sodium phosphate solution (50 mL, 5 mM, pH 6.7) and resuspended in acidic sodium chloride (100 mM, pH 2.0, 4 °C, 2 h). The cell suspensions were then recentrifuged (8000× *g*, 10 min, 4 °C), and each cell-free supernatant (0.5 mL) was applied to a C18 reversed-phase SPE column (Sep-Pak Plus, Waters Corporation, Milford, MA, USA) washed with acetonitrile 50% and preequilibrated with 10 mL of 0.1% formic acid. The column was washed with 10 mL of 0.1% (*v*/*v*) formic acid, and the bacteriocins were eluted with 5 mL of 50% (*v*/*v*) acetonitrile. The acetonitrile-containing eluents were evaporated in a water bath at 39 °C before testing the bacteriocins for activity, susceptibility to proteases and reducing agents, and thermal stability.

The purified peptides were separated by Tricine-sodium dodecyl sulfate (SDS)-polyacrylamide gel electrophoresis (PAGE) (16% acrylamide gel) using the method of Schagger [45] and following instructions from Bio-Rad bulletin 6040. The gel was fixed in 20% methanol/10% acetic acid (*v*/*v*) for 20 min, sensitized with 0.2% sodium thiosulfate (Na_2_S_2_O_3_) for 1.5 min, washed in deionized water, and stained with 0.4% silver nitrate for 20 min. Next, the gel was washed again with deionized water and developed in a solution of 6% sodium carbonate and 0.02% formaldehyde. Development was stopped by incubating the gel in 5% acetic acid (*v*/*v*) for 5 min.

The bacteriocin eluents were treated with proteases (7 U Pronase E or 15 U trypsin, mL^−1^) for 1 h or with reducing agents (β-mercaptoethanol 10%, and DTT 10 mmol/L in a 1:1 ratio with each peptide) for 3 h and activity was assessed by adding these treated fractions (20 µL) to agar wells (5 mm diameter) in BHI agar plates that had been inoculated with 40 µL of *Lactococcus lactis* ATCC 19435 (an indicator organism that was highly susceptible to all tested peptides) with OD_600 nm_ adjusted to 0.5 of the McFarland Standard. To assess the thermal stability of the peptides, bacteriocin eluents were autoclaved at 100 °C/20 min and tested for antibacterial activity as described above. The agar plates were incubated aerobically at 37 °C and bacteriocin activity was assessed from the size of the zone of clearing.

## 3. Results

### 3.1. Isolation and Characterization of Ruminal Streptococci

Four hundred and sixty-three (463) isolated colonies that grew on a semi-synthetic medium in agar plates were obtained from ruminal contents of Nelore steers. One hundred and six (106) isolates showing inhibitory activity against at least two bacterial targets were initially characterized based on phenotypic traits (colony morphology, cell arrangement, and Gram-staining) (Table S1). All 106 isolates stained Gram-positive and 81 isolates showed spherical to ovoid shapes (cocci) arranged in chains (streptococci), whereas 25 isolates were rod-shaped, occurring in pairs (diplobacilli) or in chains (streptobacilli). These isolates were catalase-negative and produced lactic acid as the major product of glucose fermentation. Overall, *Escherichia coli* ATCC 10536 (*n* = 82) and *Lactobacillus paracasei* subsp. *paracasei* ATCC 355 (*n* = 68) were the most susceptible organisms in the deferred inhibition assays (Table S1). None of the ruminal streptococci showed inhibitory activity against the drug-resistant *Acinetobacter baumanni* ATCC 19606 under the conditions tested in this study. Five streptococcal isolates showing higher inhibitory activity against a broader range of bacterial targets were initially characterized by sequencing the 16S rRNA gene and selected for whole-genome sequencing and genomic characterization (Table 1).

### 3.2. Streptococci Phylogeny

**Figure 1:**
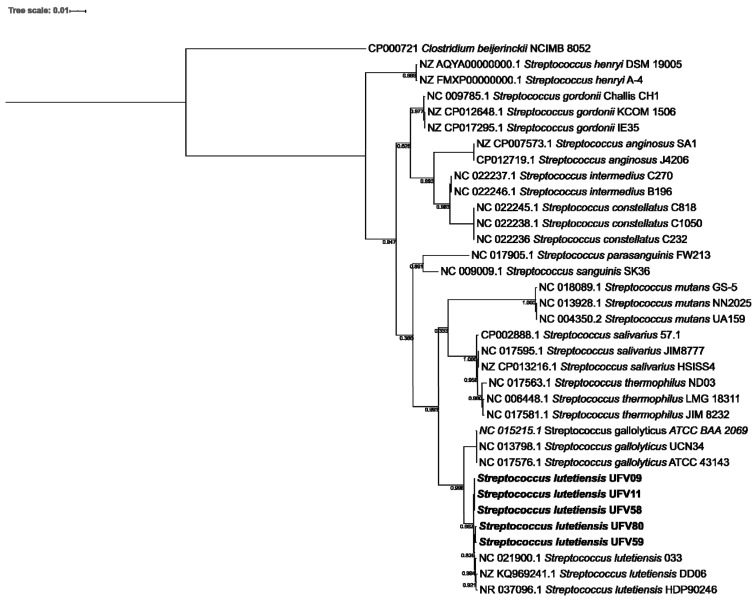
Phylogenetic tree based on 16S rRNA gene sequences from ruminal streptococci isolated from Nelore steers and other *Streptococcus* spp. The 16S rRNA gene sequences were aligned by RDP Aligner release 11 and the tree was reconstructed using the Maximum Likelihood method with FastTree v2.1 (1000 replicates) and visualized and annotated in the iTOL platform. The bootstrap values are shown near resolved branches. The scale bar represents 0.01 nucleotide substitutions per nucleotide position.

Multiple alignments of the 16S rRNA gene sequences from the ruminal streptococci selected in the current study (see above) and a subset of 29 publicly available *Streptococcus* spp. indicated that the ruminal streptococci formed a monophyletic clade with members of the *Streptococcus lutetiensis* group (Figure 1). The distances between the *S. lutetiensis* and *S. gallolyticus* strains were relatively small, which is in agreement with their capacity to inhabit the gastrointestinal tract (GIT) of animals and humans. Also, the BLASTn searches in the 16S rRNA gene sequences of the GenBank database (http://blast.ncbi.nlm.nih.gov/Blast.cgi, accessed on 14 June 2021) exhibited nucleotide identities from 99.86% to 100% between *Streptococcus lutetiensis* HDP90246, *Streptococcus lutetiensis* DD06, and *Streptococcus lutetiensis* 033 (Accession number: NR_037096.1, NZ_KQ969246.1, and NC_021900.1, respectively) with the query coverage of at least 94% and a minimum alignment score of 2708.

### 3.3. General Features of the Ruminal S. lutetiensis Genomes

**Figure 2:**
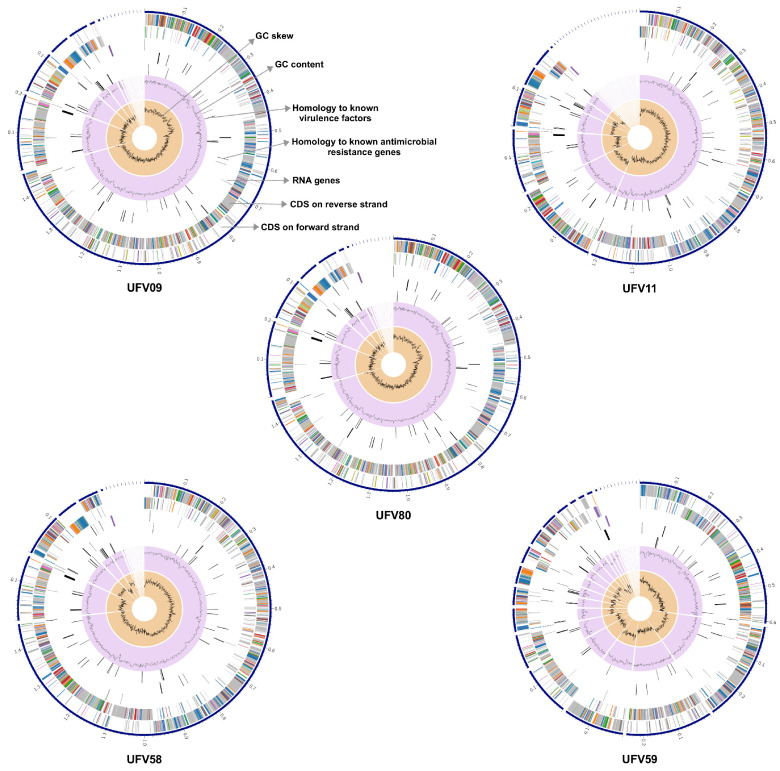
Circular genome maps of the ruminal *Streptococcus lutetiensis* strains displaying relevant genome features. The first (outermost) blue line shows the contigs; The second circle shows open reading frames oriented in the forward direction, whereas the third circle indicates those oriented in the reverse direction; the fourth circle shows the tRNA genes and the rRNA operons; The fifth and sixth circles show genes with homology to known antimicrobial resistance genes and known virulence factors, respectively; the seventh circle (purple background) shows the GC content (%) plot and the innermost circle (orange background) shows the GC skew. The colors of the open reading frames in the forward and reverse direction indicate the subsystem these genes belong to (see Subsystems details below). Empty spaces represent absence of sequence homology to each respective feature layer. The concentric circles showing different features of the *S. lutetiensis* genomes were generated using the Circos program.

The genome sizes of the ruminal streptococci isolated in the current study ranged from 1.84 (UFV09) to 1.97 Mbp (UFV59). Relevant genome features and the distribution of specific genomic regions are represented in graphical genome maps (Figure 2). The orientation of the open reading frames (ORFs) varied among the *S. lutetiensis* genomes, being more discontinuous in the genome of *S. lutetiensis* UFV59 than in the genomes of isolates UFV09 and UFV80 where two continuous blocks of gene orientation were observed. Genes encoding for RNA, antimicrobial resistance, and virulence factors were identified, and were randomly distributed within the genomes of the ruminal *S. lutetiensis* strains. Although the GC content was similar across the genomes, the GC skew showed substantial shifts in the GC contents as revealed by abrupt polarity changes in the nucleotide skew plots of strains UFV59 and UFV11.

The general genome features of all five isolates are summarized in Table 2. The number of contigs ranged from 16 (UFV58) to 33 (UFV11) and the N50 ranged from 1,214,823 bp (UFV09) to 248,939 bp (UFV59). The average GC content of the genomes was 37.8% and none contained plasmids. *S. lutetiensis* UFV09 showed the smallest genome (1,849,765 bp), while the largest belonged to *S. lutetiensis* UFV59 (1,977,774 bp). These genomes were predicted to contain a total of 1793 and 1903 protein-coding genes, respectively, with ~82% of those having predicted functions and at least 49% being classified into protein subsystems by the PATRIC platform. The *S. lutetiensis* chromosomes contained 48 (UFV59) to 51 (UFV58) tRNA genes and about 5 rRNA operons CRISPR-Cas system elements were exclusively found in the UFV80 genome. Sequences matching antibiotic resistance genes and virulence factors were retrieved from the genomes of all ruminal streptococci according to the analyses performed using PATRIC_VF, VFDB, and Victors database. Overall, 111 antibiotic resistance genes were identified in the chromosomes of all five ruminal *S. lutetiensis* strains. Most of these genes (80) were classified as homologs of 15 proteins (Alr, Ddl, Dfr, EF-G, EF-Tu, FolP, GyrA, GyrB, Iso-tRNA, KasA, MurA, RpoB, RpoC, S10p, and S12p) reported as antibiotic targets in susceptible cells. Additionally, 78 genes coding for membrane transporter proteins were predicted in the genomes of the ruminal *S. lutetiensis* strains. Sixty-one of these genes were functionally classified as ATP-binding cassette (ABC) superfamily, 10 genes as PTS mannose-fructose-sorbose (man) family, five genes as cation channel-forming heat shock protein-70 (hsp70) family, one gene as iron/lead transporter (ilt) family, and one gene showed homology to the outer membrane lipopolysaccharide export porin (lps-ep) family.

### 3.4. Analysis of the Predicted Proteomes from Ruminal S. lutetiensis

**Figure 3:**
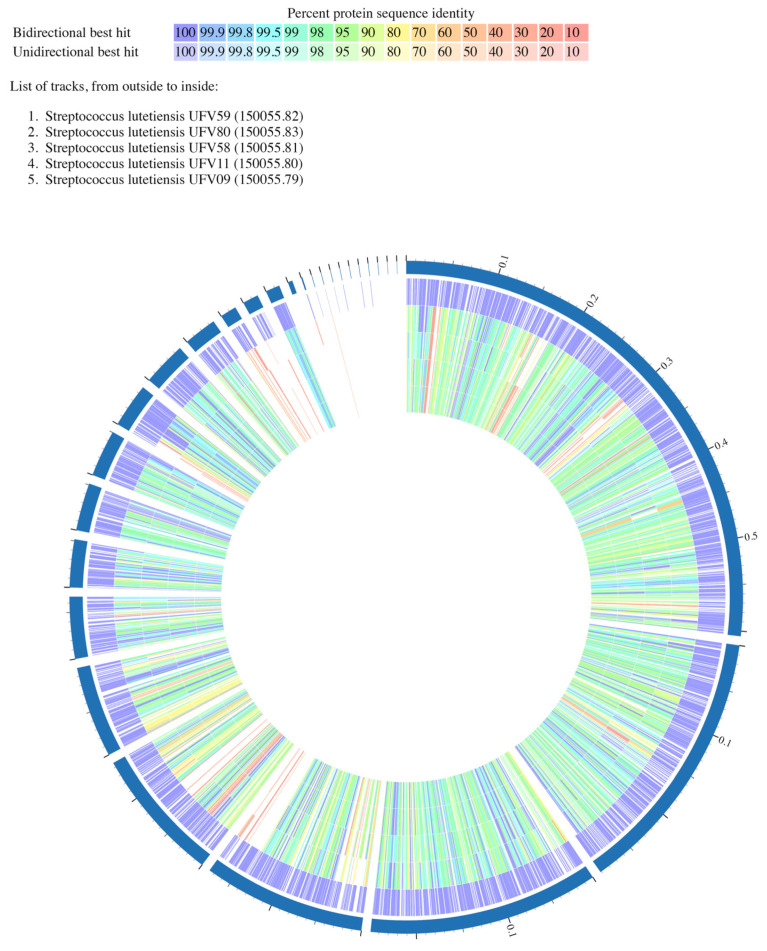
Circular representation of protein conservation in the ruminal *Streptococcus lutetiensis* genomes. The protein comparisons were performed using the bidirectional BLASTp in the PATRIC platform. *S. lutetiensis* UFV59 was used as the reference genome.

The predicted protein sequences of the five *S. lutetiensis* genomes were analyzed by the bidirectional BLASTp. This reciprocal comparison, an analysis often used to infer gene orthologs, classified the best hits by the match occurrence (unidirectional and bidirectional) and by protein percent identity (Figure 3 and Table S2). For this, the chromosome of *S. lutetiensis* UFV59 was used as reference (the largest genome among the *S. lutetiensis* strains sequenced in this study containing 1903 CDS) and the non-common best hits (unidirectional) and best common hits (bidirectional) were listed (Table S2). The sequence coverage matches ranged from 30% to 100%. The proteome comparison identified 1751 protein sequences in *S. lutetiensis* UFV59 that were present in at least one of the compared genomes. Most of the matched protein sequences (1665) were found in all five genomes while the remaining protein sequences (85) were randomly distributed across the compared genomes. From the protein sequences distributed in all five genomes, 96.9% (1615) were classified as bidirectional. Among these, 1486 protein sequences showed identities higher than 90%. Only 40 sequences (2.4%) were classified as unidirectional and their protein percent identity ranged from 20% to 94%. Additionally, exclusive protein sequences (not shared with the other genomes) were also observed in all five *S. lutetiensis* strains distributed as follows: nine proteins in UFV09, 21 proteins in UFV11, 82 proteins in UFV58, 152 proteins in UFV59, and 149 proteins in UFV80. Interestingly, all nine exclusive proteins found in strain UFV09 were localized in the same genomic region.

### 3.5. Functional Analysis of the Streptococcus lutetiensis Genomes

The predicted genes of all ruminal *Streptococcus lutetiensis* genomes were classified into 11 superclasses and 22 classes (Figure S1). Approximately 41% of all protein-coding genes were clustered into the metabolism superclass, while protein synthesis represented the highest group within the class, containing ~18% of the predicted genes. The majority of the proteins classified in the phosphate metabolism group were identified as phosphate ABC transporters or regulatory proteins in the genomes of *S. lutetiensis*. The phosphate sensory protein (PhoR) was only found in *S. lutetiensis* UFV59. The genome of strain UFV59 also showed twice as many genes related to phosphate metabolism (14 genes) compared to the other strains. The open reading frames of these genes were oriented in both coding directions in *S. lutetiensis* UFV59, but the same was not observed in the genomes of other strains.

### 3.6. Analysis of CRISPR/Cas9 Systems in the Genomes of Ruminal S. lutetiensis

**Figure 4:**
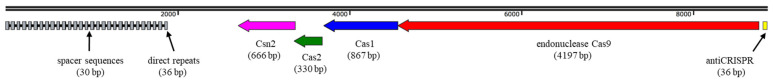
Schematic representation of the CRISPR-Cas9 (Type II-A) array system identified in the *Streptococcus lutetiensis* UFV80 genome. The arrows indicate the direction of transcription for each gene. The entire array sequence covered a region of 8850 bp in the bacterial genome.

The genome of *Streptococcus lutetiensis* UFV80 harbored a putative CRISPR-Cas9 system (Type IIA) (start position: 805,314/end position: 814,163) encoded in the forward DNA strand and containing the following elements: antiCRISPR sequence (36 bp, 33% GC), four cas genes: endonuclease Cas9 (4197 bp, 31% GC), Cas1 (867 bp, 32% GC), Cas2 (330 bp, 35% GC), Csn2 (666 bp, 32% GC), 29 direct repeats sequences with the consensus sequence 5′-GTTTTGGAACCATTCGAAACAACACAGCTCTAAAAC-3′ (36 bp, 39% GC content) and 28 additional spacer sequences (30 bp, ranging from 17% to 57% GC content) (Figure 4). Only one spacer redundancy was identified between spacer 11 and spacer 14. A BLASTn-short search of 28 spacer sequences against the Hungate CRISPR spacer sequences [35] identified a positive match showing a maximum coverage of 50% and identical matches ranging between 93.3% to 100% in 41 ruminal strains (Table S3). The repeat consensus sequence analysis using CRISPRmap showed a pairwise identity value of 57.04% and the structure index consensus value of 0.79. This repeat sequence was associated with the family 27 and superclass E with the structural motifs 14 (Figure 4). BLAST searches for potential CRISPR targets identified at least one hit with a score above 20 for 21 spacers, while 6 spacers (1, 10, 21, 22, 23, and 26) did not result in any positive hits (Table S4). BLAST searches in the HuVirDB database did not return any hits for the CRISPR targets.

### 3.7. Genome Mining of Biosynthetic Gene Clusters for Bioactive Compounds

**Figure 5:**
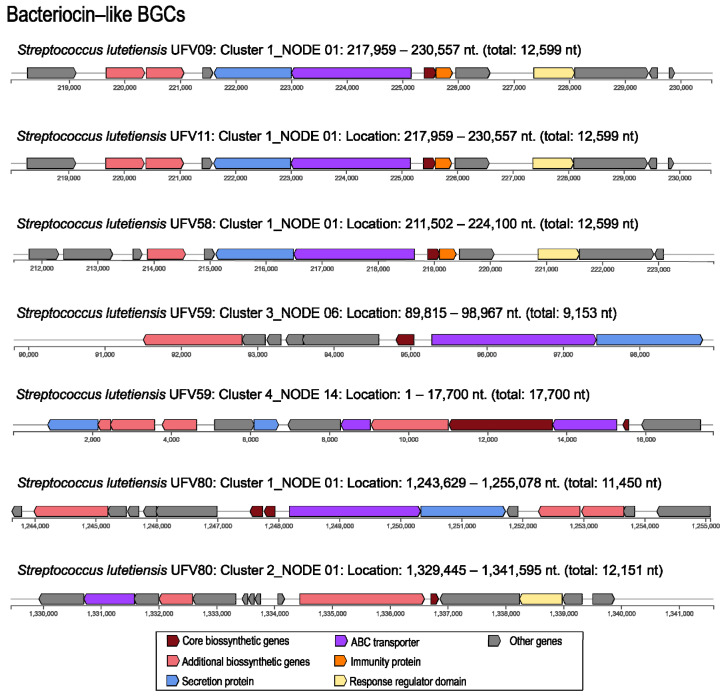
Schematic representation of biosynthetic gene clusters for bacteriocin production in the genomes of ruminal *S. lutetiensis*. The genomes were submitted to antiSMASH 5.0 analysis and the putative gene clusters for bacteriocin production were assembled using a local BLAST method and reference gene cluster. The arrows indicate the direction of transcription for each gene and the colors indicate gene function.

At least one putative biosynthetic gene cluster related to bacteriocin production was identified in all the five genomes of the ruminal *S. lutetiensis* strains using antiSMASH 5.0. The genomes of *S. lutetiensis* UFV09, UFV11, and UFV58 shared a highly conserved putative bacteriocin gene cluster, while two distinct clusters were detected in the genomes of *S. lutetiensis* UFV59 and UFV80 (Figure 5). All biosynthetic gene clusters were associated with the production of class II bacteriocins, except for cluster 4 in *S. lutetiensis* UFV59, which is associated with the biosynthesis of a class I bacteriocin (lantibiotic). Additionally, one NRPS gene cluster was detected in the genomes of *S. lutetiensis* UFV09, UFV11, UFV58, and UFV59 while one cluster associated with aryl polyene biosynthesis was detected in all ruminal *S. lutetiensis* genomes as well as for T3PKS, but only strains UFV59 and UFV80 showed one cluster associated to furan biosynthesis (Figure S2).

**Figure 6:**
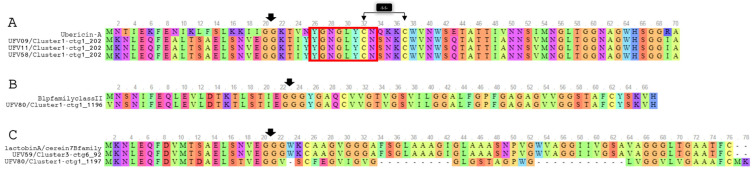
Visualization of the multiple-sequence alignment from Clustal Omega (plotted using http://msa.biojs.net/app/, accessed on 14 June 2021) of the *S. lutetienses* precursor peptides identified in this study. The colors show the conservations of the amino acid at each position of the alignments. (**A**) Precursor peptides extracted from the *S. lutetiensis* UFV09, UFV11, and UFV58 clusters showing sequence similarity with Ubericin A. The red box represents the amino acids of the conserved region (YGNGVXC) of the ‘pediocin box’ motif. (**B**) Alignment of the putative precursor peptide from cluster 1-ctg1_1196 of *S. lutetienses* UFV80, and (**C**) Alignment of the precursor peptide from cluster 1-ctg1_1197 of *S. lutetienses* UFV80 and cluster 3-ctg6_92 of *S. lutetienses* UFV59 with the lactobinA/cerein7B family. The black arrows indicate the leader peptide cleavage site and disulfide bond.

All the gene sequences of the bacteriocin clusters shown in Figure 5 were subjected to pairwise alignment using Clustal Omega (https://www.ebi.ac.uk/Tools/msa/clustalo/, accessed on 14 June 2021) and Jalview [46] for visualization (data not shown). Cluster 1 from *S. lutetiensis* UFV09 and UFV11 were identical and showed high similarity (93.03%) to the cluster 1 found in the genome of *S. lutetienses* UFV58. These sequences showed 68.57% identity with the gene cluster encoding ubericin A, a class IIa bacteriocin. Clusters 3 and 4, identified in the genomes of *S. lutetienses* UFV59 and UFV80, respectively, showed homology of 89.31% in pairwise alignments. Alignments based on BLASTp similarity showed that precursor peptides from the clusters 1 and 2 of *S. lutetiensis* UFV80 had 98% and 95% similarity, respectively, with the Blp family class II bacteriocin (NCBI Reference Sequence: WP_074481142.1) while the precursor peptide from the cluster 1 of *S. lutetiensis* UFV59 was identical to the lactobin A/cerein 7B family (NCBI Reference Sequence: WP_074602958.1). The precursor peptides identified using antiSMASH were aligned with the most related known pre-peptides based on the BLASTp results (Figure 6). The bactericins identified in the genomes of *S. lutetiensis* UFV09, UFV11, and UFV58 were highly similar to ubericin A, but specific differences were noted in the predicted precursor peptides. Two other precursor peptides predicted in the genomes of *S. lutetiensis* UFV59 (Cluster4-ctg14_12) and *S. lutetiensis* UFV80 (Cluster2-ctg1_1282) showed low similarity with class II-like bacteriocins from the BLASTp analysis. Taken together, these results indicate that the AMPs produced by the ruminal *S. lutetiensis* isolates represent novel antimicrobial peptides.

**Figure 7:**
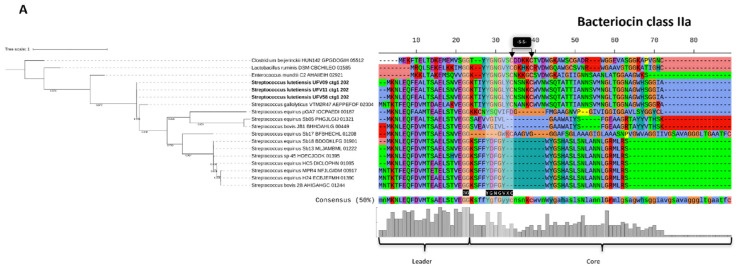

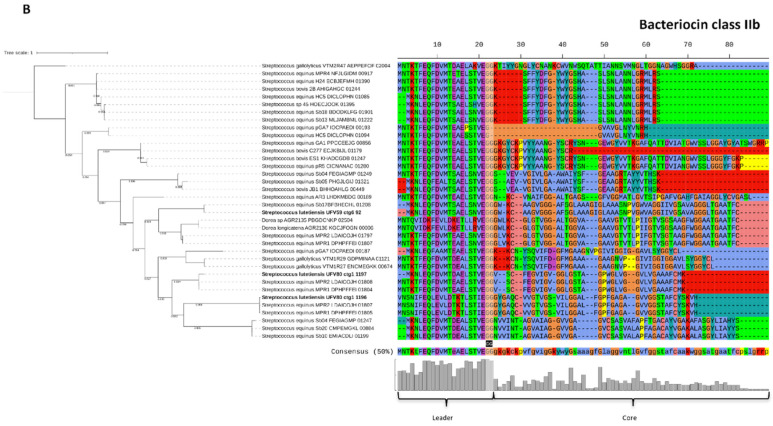
Phylogenetic tree and alignment of precursor peptides predicted in the genomes of *S. lutetienses* strains with the genomes of ruminal bacteria from the Hungate1000 project. Phylogenetic tree of the bacteriocin class IIa (**A**) and class IIb (**B**). Sequences were aligned using Muscle v3.8.31 and the phylogenetic trees were constructed using FastTree v2.1 (Maximum Likelihood method, 1000 replicates). Trees were visualized and annotated in the Interactive Tree Of Life v5 (iTOL). The bootstrap values are shown near resolved branches. The scale bar represents 0.01 nucleotide substitutions per nucleotide position. The leader and core regions of the precursor peptides are indicated below the conservation annotation sequence by the black brackets. *S. lutentienses* strains characterized in this study are shown in bold. Conserved amino acid residues related to the bacteriocin are highlighted by hatched area and black arrows.

The occurrence of similar precursor peptide sequences found in the five ruminal *S. lutetiensis* genomes was evaluated by BLASTp search against the annotated genomes of the Hungate1000 project. This analysis revealed the presence of similar class II bacteriocin precursor peptide sequences distributed in 29 ruminal genomes, with the majority associated with members of the genus *Streptococcus* (Figure 7). The percentage of identical matches and the bit score ranged between 39.8% to 100% and 66.6 to 104, respectively. The ctg14_12 precursor peptide of cluster 4 from the *S. lutetienses* UFV59 did not show any matches while the ctg1_1280_precursor peptide from cluster 2 in the genome of *S. lutetienses* UFV80 showed only two matches with the *Streptococcus gallolyticus* VTM2R47 and *Streptococcus bovis* ATCC 33317 genomes. Multiple sequence alignment confirmed the presence of conserved amino acid sequences previously associated with biochemical characteristics of each bacteriocin class. The leader peptide portion showed higher sequence conservation in both bacteriocin class alignments when compared to the core peptide.

### 3.8. Characterization of the Bacteriocins Produced by Ruminal S. lutetiensis

Preliminary experiments indicated that *Streptococcus lutetiensis* UFV9, UFV11 and UFV58 were the only cultures showing antimicrobial activity in liquid media under the growth conditions used in the current study. These strains had cell-associated bacteriocins that could be liberated from the cells using acidic NaCl, as described for other ruminal streptococci [47]. The activity of the purified peptides produced by *S. lutetiensis* UFV9, UFV11 was confirmed against some of the strains that were originally susceptible in the agar overlays (Table S5). For *S. lutetiensis* UFV58, bacteriocin recovery was low and activity could be demonstrated only against *Lactococcus lactis*. The proteinaceous nature of these molecules was confirmed by the inactivation of their antibacterial activity using trypsin and/or pronase E (Figure S3a). Additionally, eluents containing the bacteriocins from all three *S. lutetiensis* strains retained their antibacterial activity after autoclaving at 121 °C/20 min, which is in agreement with the expected thermal stability of these antimicrobial peptides. To evaluate if reducing agents could affect antimicrobial activity, the peptides from *S. lutetiensis* UFV09 and UFV11 were treated with β-mercaptoethanol and DTT (1:1 ratio), but the activity after 3 h of incubation was comparable to the control (1:1 ratio peptide/MiliQ water) (Figure S3b). The SDS-PAGE also confirmed our in silico results and demonstrated that the bacteriocins predicted in the genomes of *S. lutetiensis* UFV09, UFV11, and UFV58 had identical molecular mass (5197 Da) (Figure S4), thus providing experimental evidence that these molecules share similarity with ubericin A (MW = 5270.5 Da), a class IIa bacteriocin produced by *Streptococcus uberis*, a causal agent of bovine mastitis.

## 4. Discussion

The *Streptococcus bovis*/*Streptococcus equinus* complex (SBSEC) currently consists of a group of seven species and subspecies that stand out for their capacity to adapt in different ecosystems, resist traditional antibiotics, and withstand the host immune system [48]. Members of the SBSEC are frequently reported as colonizers of animal gastrointestinal tract (GIT) and some strains can be described as pathobionts due to their zoonotic characteristics. Nonetheless, the SBSEC is increasingly being recognized as predominant players in food fermentation [49], with many bacteria of this group being ingested regularly in fermented products of plant and animal origin as part of the human diet. These features make this bacterial complex unique among the genus *Streptococcus* for harboring both pathogenic and food-grade strains [50]. Previous reports also suggest that ruminal SBSEC members are promising sources for potentially useful antimicrobial peptides [12,51].

Here, we evaluated the antimicrobial activity of 463 fresh rumen isolates, of which 81 (17.5%) were presumptively classified as *Streptococcus* spp. showing inhibition against distinct bacterial targets. Phylogenetic analyses of the 16S rRNA gene from five isolates with a broader spectrum of activity indicated that the ruminal streptococci formed a monophyletic clade with members of the *S. lutetiensis* group showing relatively small distances to *S. lutetiensis* and *S. gallolyticus* strains. Both *S. lutetiensis* and *S. gallolyticus* belong to the SBSEC, which includes non-beta-hemolytic streptococci lactic acid bacteria typically considered as commensals or opportunistic pathogens with diverse lifestyles. Whole-genome sequencing and comparative genomic analysis of the five selected *S. lutetiensis* strains revealed many shared features essential for their survival in the rumen environment and putative genes associated with their antimicrobial activity. These isolates inhibited both human and animal pathogens in diffusion assays and genome mining predicted highly conserved biosynthetic gene clusters (BGCs) putatively encoding antimicrobial peptides in their genomes, suggesting that these antimicrobials may help them to compete with other bacteria for resources in the rumen ecosystem.

The BGCs detected in the genomes of ruminal *Streptococcus* were mostly associated with the production of class-II bacteriocins (Pfam PF01721), which appear to be distributed in other ruminal bacteria of the phylum *Firmicutes*, especially among members of the orders *Lactobacillales* and *Clostridiales* [12]. These BGCs harbored genes with a conserved protein domain of an ABC-type bacteriocin transporter (family TIGR01193) and accessory proteins related to protein efflux and processing (LagD). Moreover, the novel peptide precursors found in *S. lutetiensis* UFV9, UFV11, and UFV58 showed a high sequence identity (68.57%) with the ubericin A precursor (ubaA) (UniProt A9Q0M7), the first reported class IIa bacteriocin in the genus *Streptococcus* (*Streptococcus uberis*) [52]. Bacteriocins belonging to class IIa, such as pediocin PA-1, enterocin A, and bacteriocin 31 are heat-stable cationic peptides with molecular masses < 10 kDa that have an N-terminal consensus sequence (YGNGVXC), known as the pediocin-box [37], which was also found in the bacteriocin precursors identified in the ruminal *S. lutetiensis* strains (Figure 6). The bacteriocins produced by *S. lutetiensis* UFV9, UFV11, and UFV58 were extracted from the bacterial pellets and SDS-PAGE analysis confirmed that the purified peptides had identical molecular mass (Figure S4), which was in agreement with the predictions made in silico for the precursor and core peptide sequences.

The gene clusters of putative bacteriocins predicted in *S. lutetiensis* UFV09 and UFV11 were identical, indicating that their genomes carry the same BGC for a class IIa bacteriocin. In addition, *S. lutetiensis* UFV58 harbored a gene cluster that was highly similar (93.03%) to the BGCs of isolates UFV09 and UFV11, suggesting that this strain probably carry a variant of the same cluster. Nonetheless, some differences were observed regarding the inhibitory activity/spectrum of action between these three isolates and the bacteriocin yield recovered from cell pellets. Analysis of the BGCs in the ruminal streptococci suggests that the production of these putative class II bacteriocins is regulated by a typical two-component system, with a histidine kinase and response regulator located adjacent to the gene encoding the putative precursor peptide. A similar genetic organization was reported for other class IIa bacteriocins such as pediocin PA-1, enterocin NKR-5-3C and leucocin A [53]. The putative class II-like bacteriocins clusters identified in the genomes of *S. lutetiensis* UFV59 and *S. lutetiensis* UFV80 also showed a conserved double-glycine-type leader peptide typical of this group [53]. The presence of sequences with high similarity to the precursor peptide sequences of class II bacteriocin in strains of *Streptococcus bovis*/*equinus* complex reinforces the ecological importance of bacteriocin production within this microbial population. Besides, the sequences evaluated by BLASTp search showed that the ctg14_12, precursor peptide of cluster 4 from the *S. lutetienses* UFV59, did not show any matches against the annotated genomes of the Hungate1000 project and the ctg1_1280_precursor peptide from cluster 2 in the genome of *S. lutetienses* UFV80 showed only two matches within all the genomes analyzed. These findings also highlight the potential of the ruminal microbiota as a rich repository for novel antimicrobial compounds.

Genes encoding putative ABC-type transporters were identified in all gene clusters, together with regulatory and immunity genes (Figure 5). *S. lutetiensis* UFV59 also harbored a putative gene cluster (cluster 4) associated with the biosynthesis of a class I bacteriocin (lantibiotic) and containing homologs of the LanC-modifying enzyme (LanC-like). The class I bacteriocins are composed of peptides that show heat stability and undergo post-translational modification catalyzed by LanB and LanC or by the LanM enzyme, with the formation of lanthionine rings [54]. In addition to the genes required for processing the precursor peptide, the lantibiotic gene cluster also harbors genes conferring immunity to the producer cells and that regulates the production of the antimicrobial peptide [55]. Nonetheless, the presence of the genes encoding modification enzymes is often used as a criterion for predicting potential lantibiotic producers [56].

Comparative genome analyses indicated minimal chromosomal rearrangements and highly similar predicted proteomes (Figure 3) among the ruminal *S. lutetiensis* strains with multiple orthologous genes shared across the genomes of strains UFV9, UFV11, UFV58, UFV59, and UFV80. However, some differences were detected between the five genomes of *S. lutetiensis* analyzed, particularly in *S. lutetiensis* UFV59, which showed changes in the position and direction of the gene sequences when compared to the other strains. The presence of unique regions in each species suggests events of gene insertions mediated by horizontal gene transfer, inversions, or deletions that may have played an important role in the evolution of the strains under study [57].

Most of the proteins classified in the phosphate metabolism cluster in the *S. lutetiensis* genomes were identified as phosphate ABC transporters or regulatory proteins. Phosphorus is an essential constituent of nucleic acids, phospholipids, and other cellular constituents and is required for cellular energy metabolism [58]. Notably, *S. lutetiensis* UFV59 genome showed twice as many genes related to phosphate metabolism (*n* = 14) compared to the other four *S. lutetiensis* strains and was the only strain harboring the phosphate sensory protein (PhoR). In *Escherichia coli*, PhoR is the histidine kinase of the phosphate regulon and responds to environmental changes of phosphate levels suggesting a similar role of the homologous protein found in the genome of *S. lutetiensis* UFV59 [59].

Previous studies have shown that ruminal streptococci are well adapted to use starch and soluble carbohydrates as energy and carbon source, showing significant increases in its population in animals adapted to diets rich in concentrated foods [60]. The ability of *S. lutetiensis* isolates to use starch was related to the presence of three alpha-amylases (EC 3.2.1.1), a pullulanase (EC 3.2.1.41), and an endo-beta-1,3-1,4 glucanase (EC 3.2.1.73), with no endo-beta-1,3-1,4 glucanase. In addition, the functional analysis of the genomes indicated an abundance of genes related to the biosynthesis of cofactors, amino acids, and vitamins in agreement with the less fastidious nature of the streptococci in the SBSEC, which allows them to grow faster than the more fastidious bacteria and proliferate in a very competitive ecosystem.

A further novel finding from this study is that the genome of *S. lutetiensis* UFV80 harbors a locus of a putative CRISPR-Cas9 system (Type IIA) similar to the system initially described in *Streptococcus pyogenes* [61]. Clustered, regularly interspaced, short palindromic repeat (CRISPR) and CRISPR-associated proteins (Cas) constitute an adaptive immune system against phages and other external genetic elements. The endonuclease activities necessary for silencing foreign DNA are concentrated in a single multi-domain protein, Cas9, guided by a coprocessor of trans-activating tracrRNA and a crRNA molecule [62,63]. The genome of *S. lutetiensis* UFV80 also shows a considerable number of spacers (*n* = 28) in the CRISPR array, which is associated with the acquisition of an immune memory that protects the bacteria from invading genetic material [61]. The large number of spacer sequences detected in *S. lutetiensis* UFV80 and the low redundancy of these spacers suggest a high CRISPR activity in this ruminal strain, which could be related to improved protection against foreign nucleic acid sequences. Although there is little evidence that CRISPR-Cas systems could affect the production of bioactive molecules in lactic acid bacteria, such systems could play multiple roles in bacterial tolerance to physiological (e.g., oxidative, cell membrane) stresses thus contributing to improve cell survival in competitive ecosystems (doi: https://doi.org/10.1128/JB.02333-14, accessed on 14 June 2021). A BLASTn-short search using the 28 spacers as query sequences confirmed the presence of similar sequences in other ruminal strains, which reinforces the relevance of this adaptative immune system in the ruminal ecosystem. To our knowledge, this is the first report of a CRISPR-Cas9 system in *S. lutetiensis*.

## 5. Conclusions

In this study, we identified conserved biosynthetic gene clusters (BGCs) associated with the production of class II bacteriocins and demonstrated that the ruminal *Streptococcus lutetiensis* are potential producers of these AMPs. The genomic analyses performed revealed that the precursor peptides of the ruminal isolates showed a high sequence identity with ubericin A, an AMP produced by a strain of *Streptococcus uberis* associated with bovine mastitis. The genomes of the *S. lutetiensis* strains also harbored a plethora of genes that are related to stress response or required for the biosynthesis of amino acids, vitamins, and fatty acids. These physiological traits are potentially relevant to the survival and colonization of *S. lutetiensis* in the rumen ecosystem. Taken together, these findings highlight the importance of exploring the microbiota of ruminants for bioprospecting new antimicrobial peptides produced by prokaryotic and eukaryotic microorganisms. Further studies are warranted to biochemically characterize the AMPs identified in this work and to investigate the factors affecting the expression of the biosynthetic genes encoding these peptides under conditions simulating the rumen environment, thus providing insights into their ecological role within complex microbial communities. In addition, this is the first report of a CRISPR-Cas9 system in *S. lutetiensis* and the type II CRISPR-Cas system found in *S. lutetiensis* UFV80 could provide a new versatile tool for engineering targeted genomic locations and other biotechnological applications.

## Figures and Tables

**Table 1:** In vitro inhibitory activity of selected streptococci obtained from the bovine rumen.

	Targets							
Isolates Identification (IDs)	*Citrobacter freudii* ATCC 8090	*Enterococcus faecalis* ATCC 4083	*Escherichia coli* ATCC 10536	*Lactobacillus paracasei* subsp. *paracasei* ATCC 355	*Listeria monocytogenes* ATCC 7644	*Proteus vulgaris* ATCC 13315	*Salmonella enterica serovar* Typhimurium ATCC 14028	*Staphylococcus aureus* ATCC 29213
**UFV9**	-	-	++	-	+	-	++	++
**UFV11**	-	-	++	-	+++	-	++	+
**UFV58**	-	-	++	+	+++	-	++	-
**UFV59**	-	++	++	-	-	-	+++	-
**UFV80**	-	+	++	+	-	-	+++	++

All target bacteria were cultured in BHI medium at 37 °C and the inhibition zone sizes were determined after overnight incubation. (−) absence of inhibitory activity; (+) presence of zone of inhibition between 2 and 3 mm diameter (weak); (++) presence zone of inhibition between 3 and 6 mm diameter (good); (+++), presence zone of inhibition ≥ 6 mm diameter (strong). Results represent the average of the triplicates.

**Table 2:** Genomic features of the ruminal *Streptococcus lutetiensis* strains.

		UFV09	UFV11	UFV58	UFV59	UFV80
	Species (rRNA 16S gene sequences)	*Streptococcus lutetiensis*	*Streptococcus lutetiensis*	*Streptococcus lutetiensis*	*Streptococcus lutetiensis*	*Streptococcus lutetiensis*
	Source	Nellore rumen	Nellore rumen	Nellore rumen	Nellore rumen	Nellore rumen
Genome assembly	N of reads	261881	170826	219215	158064	166894
	Contigs	21	33	16	28	18
	L50	1	1	1	3	1
	N50	1451917	1214823	1463551	248939	1460340
	Genome Size (bp)	1849765	1854024	1854488	1977774	1902765
	GC Content (%)	38.00	38.03	38.05	37.43	37.88
	Plasmids	-	-	-	-	-
	Chromosomes	1	1	1	1	1
Genome annotation	PATRIC ID	150055.79	150055.80	150055.81	150055.82	150055.83
	CDS	1793	1808	1824	1903	1860
	tRNA	50	50	51	48	49
	rRNA	5	5	5	5	6
	crispr repeat	-	-	-	-	29
	crispr spacer	-	-	-	-	28
	crispr array	-	-	-	-	1
Protein features	Hypothetical proteins	296 (16.51%)	299 (16.54%)	322 (17.65%)	362 (19.02%)	312 (16.77%)
	Proteins with functional assignments	1497 (83.49%)	1509 (83.46%)	1502 (82.35%)	1541 (80.98%)	1548 (83.23%)
	Proteins with EC number assignments	0	0	0	0	0
	Proteins with GO assignments	473	481	474	481	477
	Proteins with Pathway assignments	0	0	0	0	0
	Proteins with Subsystem assignments	0	0	0	0	0
	Proteins with PATRIC genus-specific family (PLfam) assignments	1748	1754	1766	1858	1779
	Proteins with PATRIC cross-genus family (PGfam) assignments	1756	1767	1771	1863	1787
	Proteins with FIGfam assignments	0	0	0	0	0
Specialty genes	Antibiotic Resistance (PATRIC)	22	22	22	22	23
	Drug Target (DrugBank)	10	14	10	10	9
	Drug Target (TTD)	1	1	2	1	1
	Transporter (TCDB)	16	16	15	17	14
	Virulence Factor (PATRIC VF)	-	2	1	1	-
	Virulence Factor (VFDB)	5	6	4	5	5
	Virulence Factor (Victors)	29	32	31	32	31
	Bacteriocin cluster (antiSMASH)	1	1	1	2	2
	NRPS (antiSMASH)	1	1	1	1	-
	T3PKS (antiSMASH)	1	1	1	1	1
	Furan (antiSMASH)				1	1
	Arylpolyene (antiSMASH)	1	1	1	1	1
ENA ID	Genome accession n°	ERR5159231	ERR5159232	ERR5159233	ERR5159234	ERR5159235

## Data Availability

The sequencing data generated in this study are available from the European Nucleotide Archive (ENA) database under the accession numbers ERR5159231, ERR5159232, ERR5159233, ERR5159234 and ERR5159235 (https://www.ebi.ac.uk/ena/browser/view/ERA3313170, accessed on 14 June 2021).

## Supplementary Materials

The following are available online at https://www.mdpi.com/article/10.3390/microorganisms10030551/s1. Figure S1: Heatmap showing the functional gene annotation of ruminal *Streptococcus lutetiensis* genomes. Figure S2: Schematic representation of biosynthetic gene clusters production for NRPS, TR3PKS, furan, and arylpolyene in the genomes of ruminal *S. lutetiensis*. Figure S3a: Well diffusion assays in an agar plate showing the antimicrobial activity of the *S. lutetiensis* UFV9, UFV11 and UFV58 bacteriocins before and after treatments with proteases and autoclaving. Figure S3b: Well diffusion assays in an agar plate showing the antimicrobial activity of the *S. lutetiensis* UFV9 and UFV11 bacteriocins after 3 h of exposure to reducing agents (β-mercaptoethanol 10%, and DTT 10 mmol/L). Peptides mixed with MiliQ water in a 1:1 ratio were used as controls. Figure S4: SDS-PAGE showing the molecular mass of the bacteriocins produced by *S. lutetiensis* UFV9, UFV11 and UFV58. Table S1: Rumen bacteria from the rumen of Nelore cattle fed with tropical forages showing inhibitory activity. Table S2: In silico proteome comparison of ruminal *S. lutetiensis* strains. Table S3: LASTn-short search results showing ruminal genomes harboring the CRISPR spacer sequences found in *Streptococcus lutetiensis* UFV80. Table S4: BLAST search results showing the predicted CRISPR targets identified in the *Streptococcus lutetiensis* UFV80 genome. Table S5: Inhibitory spectra of the purified bacteriocins from *S. lutetiensis* UFV9, UFV11, and UFV58.
