# Supplementary material for: Whole-Genome Sequencing and Comparative Genomic Analysis of Antimicrobial Producing Streptococcus lutetiensis from the Rumen
Source: Microorganisms. 2022 Mar 3;10(3):551. doi: 10.3390/microorganisms10030551 (PMC8949432; doi:10.3390/microorganisms10030551)
Supplement: Supplementary file 1 [file microorganisms-10-00551-s001.zip › supplementary-proofback/Figure S2.pdf]

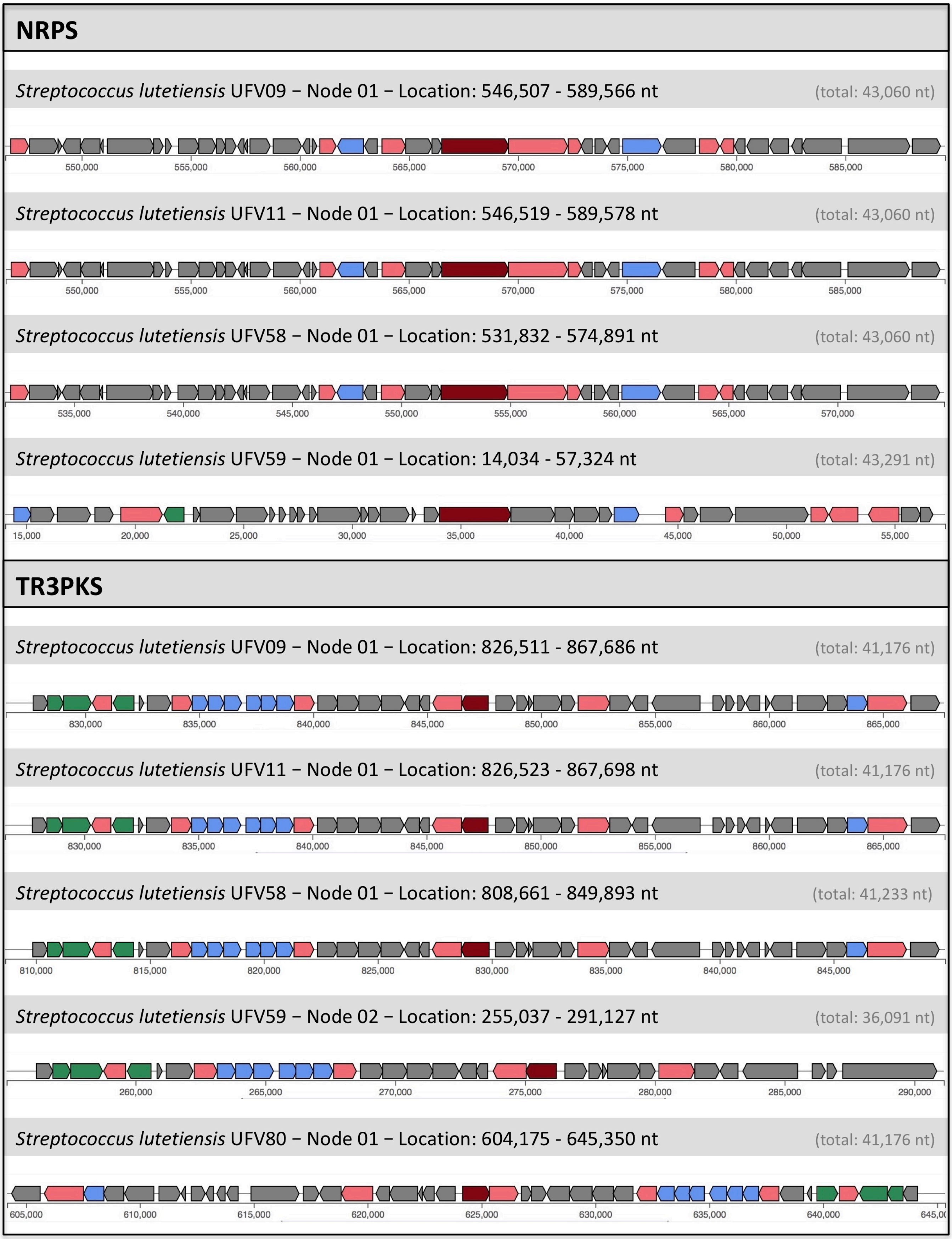
NRPS
Streptococcus lutetiensis UFV09 – Node 01 – Location: 546,507 - 589,566 nt
(total: 43,060 nt)
550,000 555,000 560,000 565,000 570,000 575,000 580,000 585,000
Streptococcus lutetiensis UFV11 – Node 01 – Location: 546,519 - 589,578 nt
(total: 43,060 nt)
550,000 555,000 560,000 565,000 570,000 575,000 580,000 585,000
Streptococcus lutetiensis UFV58 – Node 01 – Location: 531,832 - 574,891 nt
(total: 43,060 nt)
535,000 540,000 545,000 550,000 555,000 560,000 565,000 570,000
Streptococcus lutetiensis UFV59 – Node 01 – Location: 14,034 - 57,324 nt
(total: 43,291 nt)
15,000 20,000 25,000 30,000 35,000 40,000 45,000 50,000 55,000
TR3PKS
Streptococcus lutetiensis UFV09 – Node 01 – Location: 826,511 - 867,686 nt
(total: 41,176 nt)
830,000 835,000 840,000 845,000 850,000 855,000 860,000 865,000
Streptococcus lutetiensis UFV11 – Node 01 – Location: 826,523 - 867,698 nt
(total: 41,176 nt)
830,000 835,000 840,000 845,000 850,000 855,000 860,000 865,000
Streptococcus lutetiensis UFV58 – Node 01 – Location: 808,661 - 849,893 nt
(total: 41,233 nt)
810,000 815,000 820,000 825,000 830,000 835,000 840,000 845,000
Streptococcus lutetiensis UFV59 – Node 02 – Location: 255,037 - 291,127 nt
(total: 36,091 nt)
260,000 265,000 270,000 275,000 280,000 285,000 290,000
Streptococcus lutetiensis UFV80 – Node 01 – Location: 604,175 - 645,350 nt
(total: 41,176 nt)
605,000 610,000 615,000 620,000 625,000 630,000 635,000 640,000

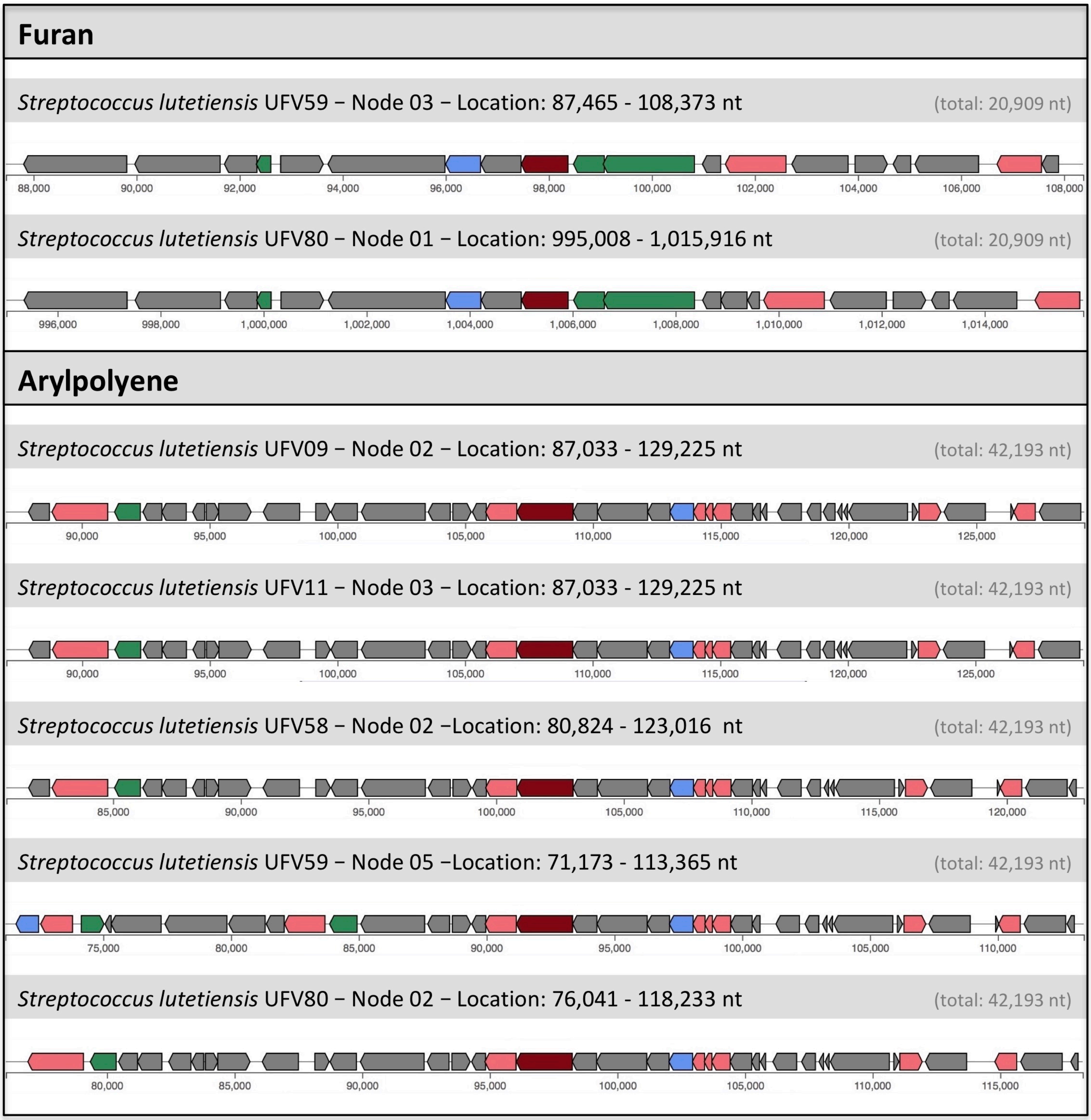
Furan
Streptococcus lutetiensis UFV59 – Node 03 – Location: 87,465 - 108,373 nt
(total: 20,909 nt)
88,000 90,000 92,000 94,000 96,000 98,000 100,000 102,000 104,000 106,000 108,000
Streptococcus lutetiensis UFV80 – Node 01 – Location: 995,008 - 1,015,916 nt
(total: 20,909 nt)
996,000 998,000 1,000,000 1,002,000 1,004,000 1,006,000 1,008,000 1,010,000 1,012,000 1,014,000
Arylpolyene
Streptococcus lutetiensis UFV09 – Node 02 – Location: 87,033 - 129,225 nt
(total: 42,193 nt)
90,000 95,000 100,000 105,000 110,000 115,000 120,000 125,000
Streptococcus lutetiensis UFV11 – Node 03 – Location: 87,033 - 129,225 nt
(total: 42,193 nt)
90,000 95,000 100,000 105,000 110,000 115,000 120,000 125,000
Streptococcus lutetiensis UFV58 – Node 02 –Location: 80,824 - 123,016 nt
(total: 42,193 nt)
85,000 90,000 95,000 100,000 105,000 110,000 115,000 120,000
Streptococcus lutetiensis UFV59 – Node 05 –Location: 71,173 - 113,365 nt
(total: 42,193 nt)
75,000 80,000 85,000 90,000 95,000 100,000 105,000 110,000
Streptococcus lutetiensis UFV80 – Node 02 – Location: 76,041 - 118,233 nt
(total: 42,193 nt)
80,000 85,000 90,000 95,000 100,000 105,000 110,000 115,000
