# Supplementary material for: Whole-Genome Sequencing and Comparative Genomic Analysis of Antimicrobial Producing Streptococcus lutetiensis from the Rumen
Source: Microorganisms. 2022 Mar 3;10(3):551. doi: 10.3390/microorganisms10030551 (PMC8949432; doi:10.3390/microorganisms10030551)
Supplement: Supplementary file 1 [file microorganisms-10-00551-s001.zip › supplementary-proofback/Table S5_R1.pdf]

Table S5. Inhibitory spectra of the purified bacteriocins from *S. lutetiensis* UFV9, UFV11, and UFV58.

Indicator organism	Inhibition zone (mm)		
	UFV9	UFV11	UFV58
Salmonella enterica serovar *Typhimurium* ATCC 14028	14	17	-
Escherichia coli ATCC 10536	14*	16*	-
Lactobacillus paracasei subsp. *paracasei* ATCC 335	13	17	-
Listeria monocytogenes ATCC 7644	-	-	-
Staphylococcus aureus ATCC 29213	-	-	-
Lactococcus lactis ATCC 19435	15	16	7

*The presence of resistant colonies were observed in the zones or clearing.
