# Supplementary figures and images for: Whole-Genome Sequencing and Comparative Genomic Analysis of Antimicrobial Producing Streptococcus lutetiensis from the Rumen
Source: Microorganisms. 2022 Mar 3;10(3):551. doi: 10.3390/microorganisms10030551 (PMC8949432; doi:10.3390/microorganisms10030551)

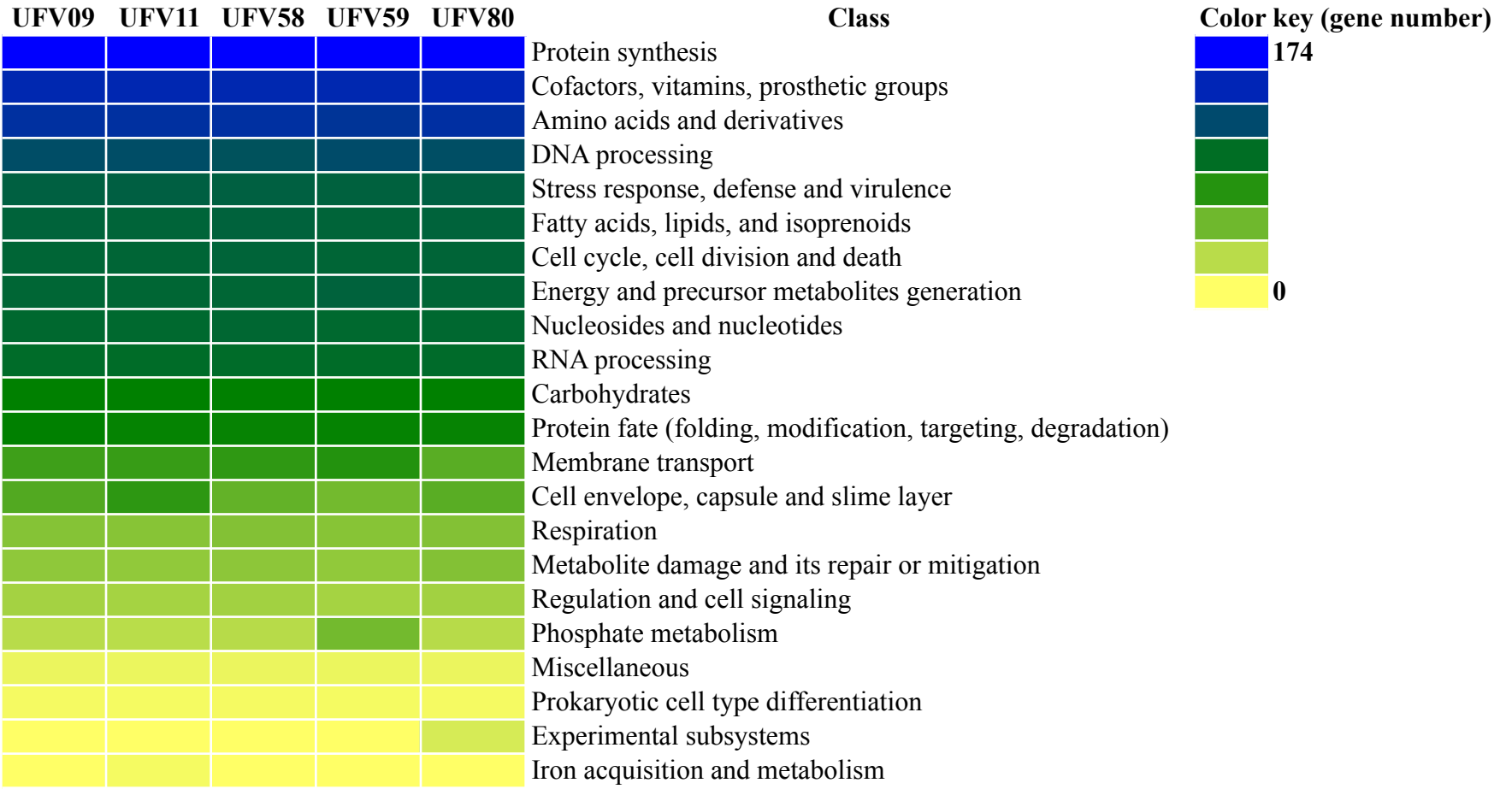

Supplement: Supplementary file 1 [file microorganisms-10-00551-s001.zip › supplementary-proofback/Figure S1.pdf]

(A)

120 °C/20 min

Pronase E

Trypsin

Eluent

9

11

58

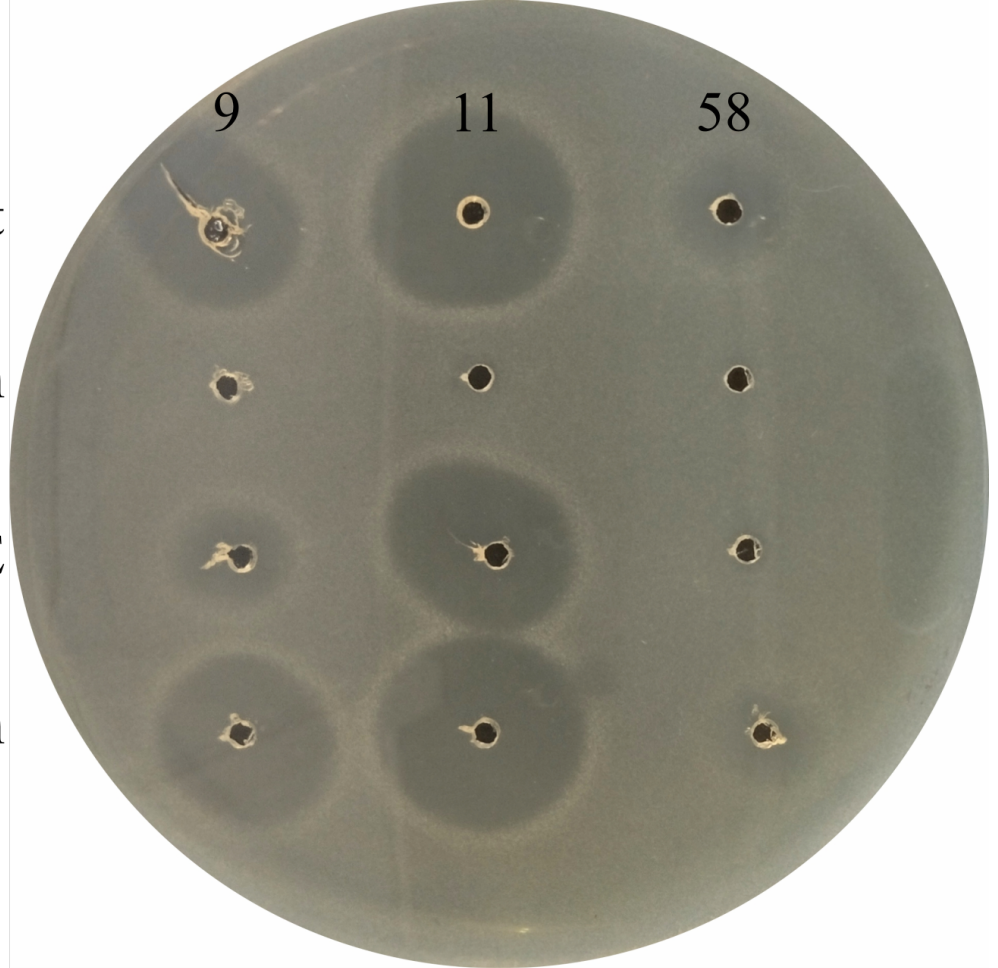

(B)

b-mercaptoethanol

DTT

Control

9

11

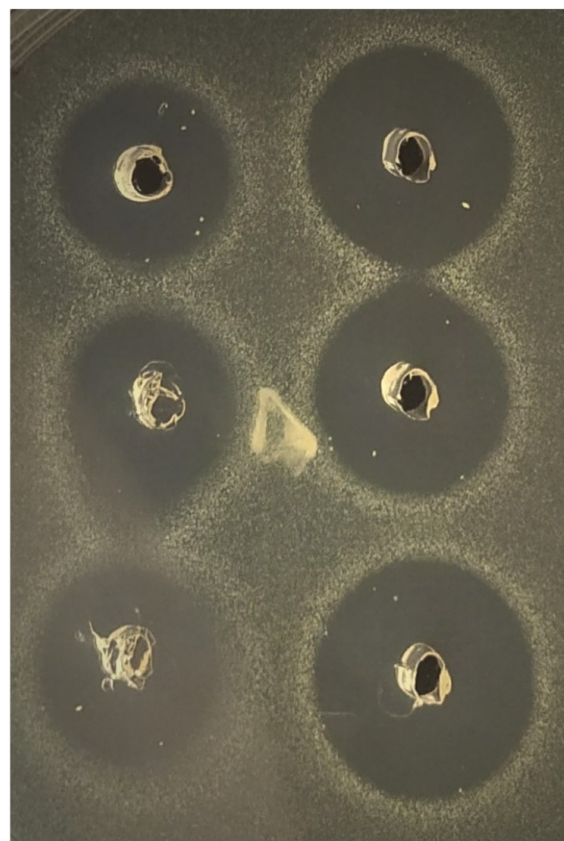

Supplement: Supplementary file 1 [file microorganisms-10-00551-s001.zip › supplementary-proofback/Figure S3_R1.pdf]

M

9

11

58

26.60

17.00

14.20

6.50

3.50

1.06

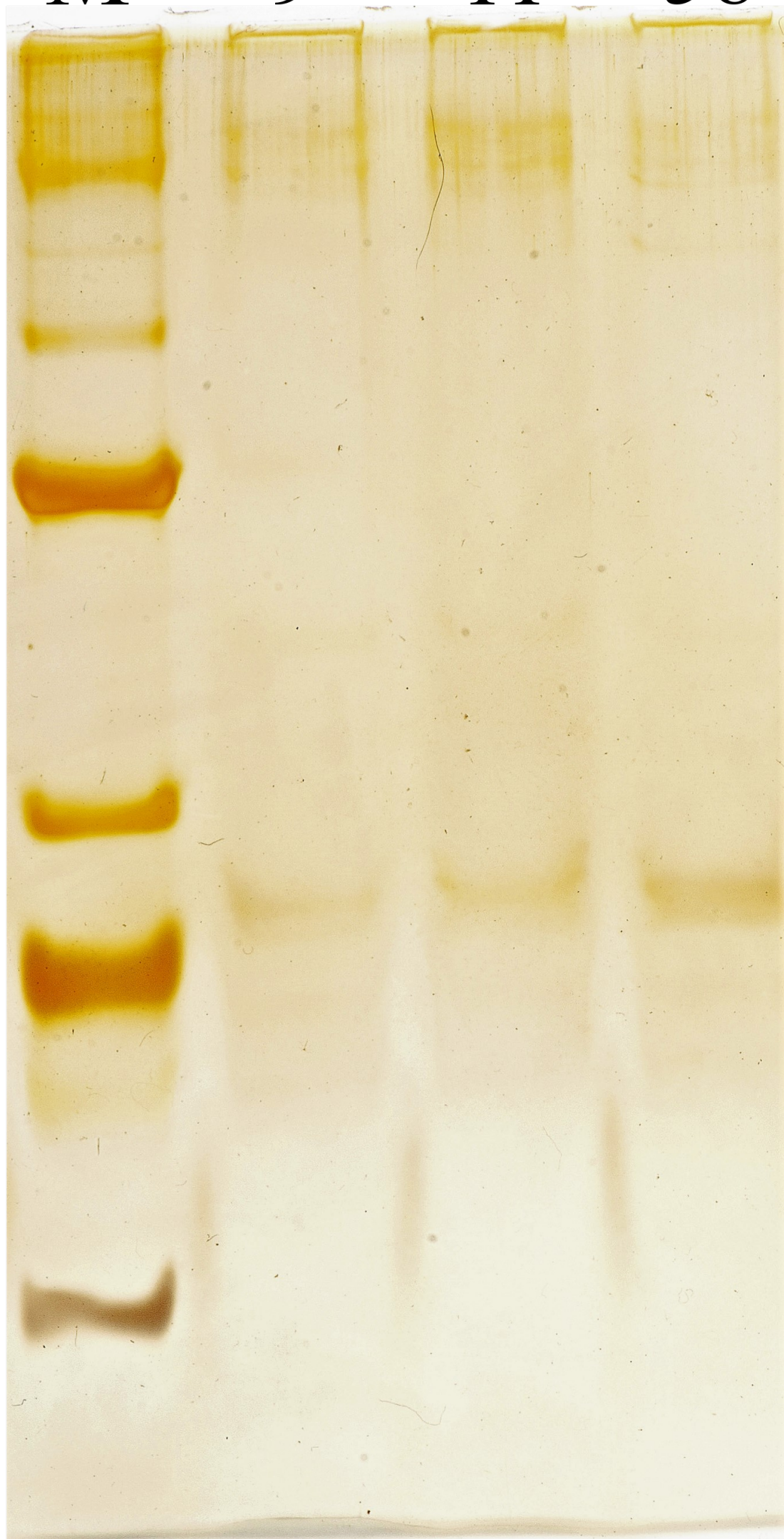

Supplement: Supplementary file 1 [file microorganisms-10-00551-s001.zip › supplementary-proofback/Figure S4.pdf]
